# Affective responses drive the impact neglect in sustainable behavior

**DOI:** 10.1016/j.isci.2023.108280

**Published:** 2023-10-20

**Authors:** Erkin Asutay, Hulda Karlsson, Daniel Västfjäll

**Affiliations:** 1 Department of Behavioral Sciences and Learning, Linköping University, 58183 Linköping, Sweden; 2 Jedi Lab, Linköping University, 58183 Linköping, Sweden; 3 Decision Research, Eugene, OR 97401, USA

**Keywords:** Environmental science, Social sciences

## Abstract

We need unparalleled human behavioral changes to mitigate the effects of climate change. However, recent studies suggest that people are not good at identifying mitigative behaviors that are effective in reducing carbon emissions. Thus, even when there is an intention to engage in climate action, people are not necessarily making the most effective choices. This suggests that there is an impact of neglecting in evaluative judgments about mitigative behaviors. Here, using an online survey (*N* = 555), we show that people have a rather poor understanding of the mitigation potential of human behaviors, and both impact judgments and the likelihood of adoption of mitigative behaviors are largely influenced by emotional processes. These findings have potential implications for how to motivate impactful climate action in the future and point toward the importance to fully understand how affect and emotions influence impact judgments and pro-environmental behavior.

## Highlights

• People like and adopt sustainable behaviors with relatively low mitigation potentials
• Sustainable behaviors evoking positive affect are perceived as impactful
• Sustainable behaviors evoking positive affect are adopted at a higher rate

## Graphical abstract

**Figure:**
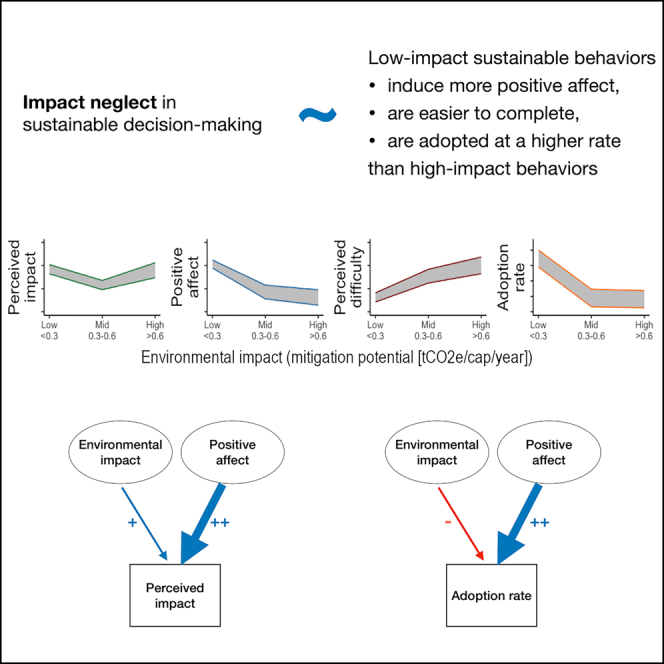

## Introduction

Unprecedented societal transitions and lifestyle changes are needed to curtail the effects of climate change. The recent IPCC reports draw attention to the potential of human behavioral changes to reduce global greenhouse gas emissions.^1^ In addition, a plethora of research studies and perspectives support the view that human behavior is central to how we mitigate the effects of climate change.^2^^,^^3^^,^^4^^,^^5^^,^^6^ Various human behaviors would reduce emissions such as consuming low-carbon products and services as well as supporting policies and technologies aimed at creating sustainable systems.^7^ Here, our focus lies on the former: mitigative human behaviors aimed at reducing emissions (e.g., purchasing low-carbon products, adopting a plant-based low-carbon diet, reducing air transport, and so forth).^1^ We argue similarly to others that instead of increasing the adoption of mitigative behaviors in general, we need to promote behaviors that are relatively more effective in reducing carbon emissions,^6^^,^^8^ and we further suggest that understanding affect and emotions as motivators of behavior will help to reach that goal.^2^^,^^9^^,^^10^^,^^11^ The current literature on the psychological antecedents of mitigative behaviors provides insights into how individual-level factors such as climate change beliefs, environmental values, social norms, and emotional reactions to climate change may increase or decrease the general engagement in pro-environmental behavior.^2^^,^^5^^,^^9^ Arguably, we need more understanding for the affective and psychological antecedents of people’s perceptions of and intentions to engage in relatively high-impact mitigative behaviors.^8^ Here, we investigate the relationship between affective reactions to mitigative behaviors, perceived impact as well as the adoption of these behaviors.

Climate change awareness and concern for its consequences are generally quite high,^12^^,^^13^ and yet, effective behavioral mitigation at scale is conspicuous by its absence.^5^^,^^14^ A partial explanation for this could be that people partake in less impactful behaviors because they have a lack of understanding that some behaviors have a higher potential to mitigate emissions than others. Indeed, people overestimate the energy used in low-energy activities (e.g., lower wattage bulbs), but greatly underestimate energy used in high-energy activities^15^ (e.g., room instead of a central air conditioner). Similarly, people underestimate the mitigation potential of some high-impact behaviors (e.g., reducing air transport) and overestimate some low-impact behaviors^14^^,^^16^^,^^17^ (e.g., recycling). Moreover, Holmgren et al.^18^ found evidence of a negative footprint illusion related to the construction of green housing, and Wynes et al.^19^ found that people have a low level of carbon numeracy. Taken together, these findings indicate that there seems to be an impact neglect in valuation judgments about sustainable behaviors. Several studies have focused on increasing knowledge about the mitigation potential of human behaviors to reduce this impact neglect, showing that an understanding for the relative impact of different behaviors can be related to an intention to adopt high-impact behaviors.^17^^,^^19^ To date, behavioral research has used information-based interventions, serving to educate people about environmental impact with for example carbon/ecological footprint calculators,^6^^,^^20^^,^^21^^,^^22^ directing the focus to climate knowledge and familiarity to understand the disconnect between impact judgments and actual mitigation potentials. However, these cognitive factors behind impact neglect are important but has been limited in scope. Surprisingly, previous investigations within the sustainability domain overlook the pervasive influence of affect and emotions on judgment and decision-making (for similar calls, see^2^^,^^9^^,^^11^). Based on the vast literature on emotion and decision-making from other decision domains ^e.g.,^^23^^,^^24^^,^^25^^,^^26^^,^^27^, we argue that affect and emotion are potential mechanisms driving the impact neglect; and to increase impactful behavioral mitigation, we need to understand the interplay between affective processes, perceived impact, and the adoption of mitigative behaviors.

Affect is an experiential system that provides information we can use in valuation judgments.^28^^,^^29^ Affect experienced as a feeling state can influence judgments by emphasizing a positive or a negative quality of an event or a behavior.^26^ This phenomenon, called affect heuristic, shows how affect guides judgments and decisions by forming easily accessible positive and negative mental tags. In a separate line of research, it has been reported that both anticipated and experienced positive affect resulting from acting “green” is an important driver of climate action.^30^^,^^31^^,^^32^ Thus, the role of affect in guiding judgments and motivating pro-environmental behavior may be seen at the mitigative behavior level. The within-person differences in affective reactions associated with different behaviors can at least partly account for the impact neglect. For instance, positive affect associated with a relatively low-impact behavior such as recycling may increase its perceived impact. It may feel good to recycle because the person is satisfied for doing their part in tackling climate change,^33^ which strengthens the positive affect associated with the action forming a positive feedback loop ensuring the sustained behavior. Hence, positive affect becomes both an antecedent and a consequence of environmental action creating a reinforcement mechanism for the behavior.^2^^,^^11^^,^^34^ However, existence of a strong affect-behavior loop for a low-impact behavior may also decrease the likelihood of adopting a high-impact behavior. Further, anticipated positive feelings resulting from pro-environmental behavior have been shown to motivate mitigative behaviors that are easy to implement,^35^ which often correspond with low-impact behaviors. Thus, many people may be reinforced in an ineffective pro-environmental behavioral pattern by positive affective experiences from undertaking easy and less impactful mitigative behaviors. Currently, we lack definitive empirical evidence for this mechanism as well as how such a mechanism could be utilized to promote the adoption of high-impact behaviors. Thus, it is crucial to understand how people’s affective reactions influence their impact perception and engagement with sustainable behaviors.

The current research looking into the relationship between affective reactions, impact perception, and pro-environmental behavior increases our understanding of the relationship between impact judgments and actual mitigation potentials through an affective lens. Our study complements the current literature, which often focuses on the individual-level factors such as emotional reactions to climate change and environmental values. We extend this literature by focusing on differences in affective reactions associated with mitigative behaviors from various domains such as food, transport, housing, energy use, and consumption. We also consider that the use of such a wide range of human behaviors contributes to our current understanding of how people’s impact perceptions of and affective reactions to sustainable behavior influences the adoption of these behaviors. We show that the mitigative behaviors that are perceived to be easy to implement and feel good to complete more often have a relatively low impact. These results have potential implications for how to motivate impactful pro-environmental behavior, instead of designing knowledge-based interventions, we may need to aim to change the affective patterns that are reinforcing low-impact behaviors at the cost of effective behavioral mitigation.

## Results

We explored the affective antecedents of impact judgments and adoption of mitigative behaviors with an online survey (N = 555, 281 women, 272 men, 1 non-binary, 1 did not disclose; age = 38.6, sd = 13.4; for descriptive statistics, see Table S1). We have selected 38 different behaviors from various domains such as food (e.g., adopting a vegan diet), transport (e.g., reducing air transport), housing (e.g., reducing living space), energy use (e.g., shifting to renewable electricity), and consumption (e.g., purchasing fewer and durable items). The selection was based on a recent meta-analysis by Ivanova and colleagues^36^ (for a full list, see Table S2), in which the researchers present a systematic review of behaviors across various consumption domains in terms of estimated reduction in carbon emissions per capita per year. Here, we use these as a measure of the objective impact to be compared with subjective impact judgments. Therefore, we ask participants to rate the perceived impact of each behavior. Moreover, we assessed affective reactions to mitigative behaviors reported on a five-point bipolar scale ranging from very negative to very positive. Participants also rated how difficult they think it would be to adopt each behavior, and reported whether they currently undertake them.

Participants then rated to what extent they feel negative (anxiety, distress, fear, guilt, shame, and worry) and positive (determination, hope, inspiration, pride, and strength) emotions in response to climate change (will be referred to as positive and negative climate emotions), which reflect the extent of the positive and negative emotional engagement with climate change. In addition, we assessed risk perceptions related to climate change, indicating how big of a risk they think climate change poses to self and others. As further control variables, participants reported the extent of their belief in the human-caused climate change (i.e., climate change belief) and their belief about the efficacy of human behaviors in reducing climate change (i.e., outcome efficacy belief).

### People have a poor understanding of the relative impact of mitigative behaviors

**Figure 1:**
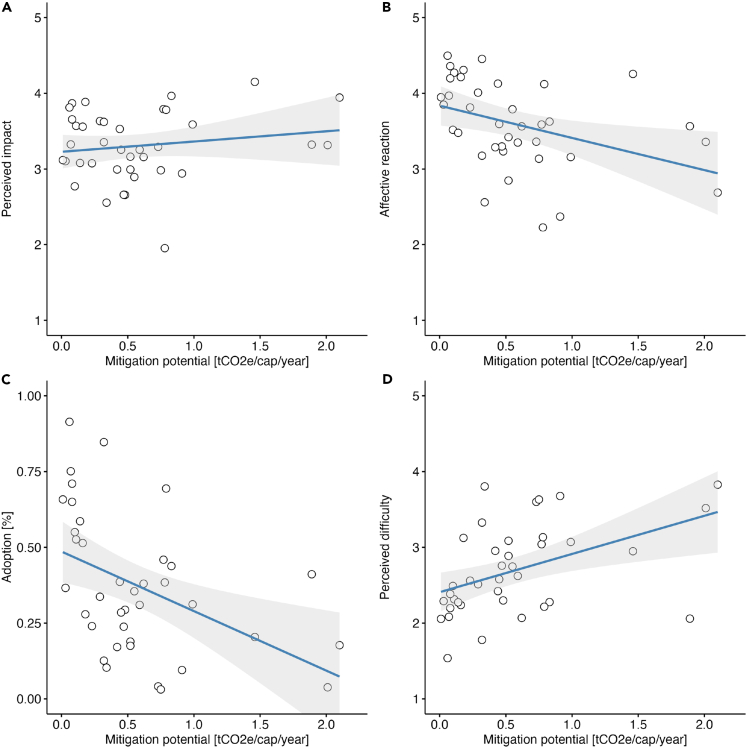
Correlations between the mitigation potentials and subjective ratings of sustainable behaviors The scatterplots show how actual mitigation potentials are related to perceived impact (*ρ = 0.04,* p *= 0.8*; panel A), affective reactions (*ρ = -0.52,* p *< 0.001*; panel B), adoption rate (*ρ = -0.5,* p *= 0.001*; panel C), and perceived difficulty (*ρ = 0.5,* p *= 0.002*; panel D). Shaded areas indicate 95% confidence intervals.

We studied Spearman rank correlations between the actual mitigation potentials and subjective ratings associated with each behavior (perceived impact, affective reactions, and perceived difficulty) as well as the proportion of participants undertaking each action to assess how objective mitigative potentials relates to subjective ratings and the adoption rates (for a full list, see Table.S2; for correlations see Figure S1). We found that perceived impact was not significantly associated with mitigation potentials (Spearman’s ρ = 0.04, p = 0.8, N = 38; Figure 1A); and the behaviors with higher mitigation potentials were rated as more difficult (Spearman’s ρ = 0.5, p = 0.002, N = 38; Figure 1D), generated more negative affect (Spearman’s ρ = −0.52, p < 0.001, N = 38; Figure 1B), and were adopted by fewer individuals (Spearman’s ρ = −0.5, p = 0.001, N = 38; Figure 1C). Instead, perceived impact was significantly associated with positive affect (Spearman’s ρ = 0.62, p < 0.001, N = 38) and a higher adoption rate (Spearman’s ρ = 0.35, p = 0.031, N = 38).

To investigate the relationship between subjective impact judgments and objective mitigation potentials at the individual level, we calculated Spearman rank correlations between perceived impact and the actual mitigation potentials for each individual. This individual coefficient can range from −1 to 1; a significant positive correlation indicates that the participant’s impact judgments are in line with the mitigation potentials. The individual coefficients distributed almost evenly around zero (53% had a positive coefficient, median correlation = 0.017, sd = 0.232), with only 10% (N = 55) of participants having a significant positive correlation between their impact judgments and the actual mitigation potentials (see Figure S1). In addition, impact judgments of 38 individuals (6.8%) were inversely correlated with mitigation potentials. Taken together, these findings indicate that (1) people have a poor understanding of the relative effectiveness of human behaviors in reducing carbon emissions, (2) they tend to adopt and like behaviors with relatively low impact, (3) they tend to think that the behaviors they like and adopt are impactful, and (4) they find behaviors with high-impact difficult to adopt in their current living situations. Interestingly, at the individual level, perceived difficulty in our data was not associated with income (Spearman’s ρ = 0.06, p = 0.17, N = 555), instead, it was associated with a lack of a belief in outcome efficacy (Spearman’s ρ = −0.33, p < 0.001, N = 555; see Figure S2 for a full correlations table over individuals). This suggests that difficulty judgments may not be a direct consequence of monetary resources, which warrants further investigation.

### Affective reactions to mitigative behaviors and climate emotions determine perceived impact

We analyzed the predictors of perceived impact using mixed-effects linear regression models. We first formulated a model with behavior ratings (perceived difficulty, affective reactions) and the mitigation potentials (Model 1 in Table.1). The standardized coefficient estimates indicated that positive affect had a robust influence on impact judgments. The objective mitigation potentials contributed to impact judgments with a smaller positive effect. Perceived difficulty also significantly contributed to impact judgments with a small negative effect. Next, we included positive and negative emotional reactions to climate change (Model 2 in Table.1), which did not influence the estimates of behavior ratings while improving the model fit (*χ*^*2*^*(2) = 65.7,* p *< 0.001*). Both positive and negative emotions related to climate change contributed to impact judgments with significant positive estimates, indicating that individuals experiencing stronger emotions in response to sustainability issues rated the mitigative behaviors as more impactful. Interestingly, negative emotions such as worry and fear as well as positive emotions such as hopefulness and inspiration independently increased perceived impact with similar effects. Finally, we included other control variables (i.e., risk perceptions and climate change and outcome efficacy beliefs; Model 3 in Table.1), which further improved the model fit relative to Model 2 (*χ*^*2*^*(3) = 61.7,* p *< 0.001*). Only the belief in outcome efficacy significantly contributed indicating that having a belief that a change in human behavior would make a difference in reducing climate change is associated with an increased impact perception. Interestingly, risk perception and climate change belief did not influence perceived impact. Note that in our sample the belief in the human-caused climate change was very high (mean = 4.4, on a scale from 1 to 5) with little variation (83% of the sample scoring above 4), which could explain the lack of a significant estimate. The inclusion of the control variables did not influence estimates for the behavior ratings. However, the significant effect of negative climate emotions disappeared, while the effect of positive climate emotions, although still significant, became somewhat smaller. Taken together, these findings indicate that having a positive affective reaction to a mitigative behavior is the strongest predictor of its perceived impact independent from its actual impact in reducing carbon emissions. Moreover, having a strong emotional engagement with climate change is associated with higher impact judgments. The effect of negative emotions such as anxiety, worry, and distress seems to be represented by an increased belief in outcome efficacy. The effect of positive emotions such as hope, inspiration, and determination did not entirely depend on risk perceptions or beliefs about climate change and efficacy.

**Table 1:** Hierarchical linear mixed models predicting impact judgments based on behavior ratings and mitigation potentials (Model 1), climate emotions (Model 2), and individual-level control variables (Model 3)

	Model 1	Model 2	Model 3
Affective reactions	**0.35 [0.33, 0.38]**^a^	**0.35 [0.33, 0.37]**^a^	**0.35 [0.33, 0.37]**^a^
Perceived difficulty	**−0.03 [-0.05, -0.01]**^b^	**−0.03 [-0.05, -0.01]**^b^	**−0.03 [-0.05, -0.01]**^b^
Mitigation potential	**0.15 [0.04, 0.26]**^c^	**0.15 [0.04, 0.26]**^c^	**0.14 [0.03, 0.25]**^c^
Positive climate emotions		**0.12 [0.08, 0.17]**^a^	**0.09 [0.05, 0.13]**^a^
Negative climate emotions		**0.13 [0.09, 0.17]**^a^	0.03 [-0.02, 0.08]
Climate change belief			−0.01 [-0.07, 0.05]
Risk perception			0.05 [-0.01, 0.11]
Outcome efficacy belief			**0.17 [0.12, 0.22]**^a^
(Intercept)	**3.30 [3.18, 3.42]**^a^	**3.30 [3.18, 3.42]**^a^	**3.30 [3.18,3.42]**^a^
N	21090	21090	21090
N (participant)	555	555	555
N (behavior)	38	38	38
AIC	51209	51159	51120
R^2^ (fixed/total)	0.11/0.52	0.16/0.53	0.19/0.54

All continuous predictors are mean-centered and scaled by 1 standard deviation. Numbers in the brackets represent 95% confidence intervals.

a p < 0.001.

b p < 0.01.

c p < 0.05.

### Pro-environmental behavior is largely determined by positive affective reactions

We analyzed the likelihood of adopting mitigative behaviors with mixed-effects logistic regression models. The predictors were affective reactions, perceived difficulty, perceived impact, and the actual mitigation potentials (Table.2). Affective reactions and perceived impact had significant Odds Ratios (OR) above 1. While the mitigation potential and perceived difficulty had significant ORs below 1. The model accurately predicted 83% of the responses. The inclusion of individual level variables (i.e., positive and negative climate emotions, risk perceptions, and beliefs in climate change and outcome efficacy) did not improve the model significantly (*χ*^*2*^*(5) = 9.3,* p *= 0.098*), which was further supported by the unaffected goodness-of-fit measures and non-significant coefficient estimates (see Table.S3). To place it in context, the results indicate that 1 SD increase in positive affect associated with a mitigative behavior increased the likelihood of adoption by more than 100%, and a similar increase in perceived impact increased the likelihood by almost 30%, all else being equal. On the other hand, 1 SD increase in perceived difficulty is associated with a decrease in the likelihood of adoption by almost 70%. Interestingly, a higher mitigation potential was also associated with a decrease in the adoption likelihood.

**Table 2:** Logistic mixed model predicting the individuals’ likelihood of undertaking a mitigative behavior based on affective reactions and impact and difficulty judgments

	Model
Affective reactions	**2.15 [1.99, 2.32]**^a^
Perceived difficulty	**0.32 [0.30, 0.35]**^a^
Perceived impact	**1.28 [1.20, 1.36]**^a^
Mitigation potential	**0.58 [0.41, 0.82]**^b^
(Intercept)	**0.34 [0.24, 0.48]**^a^
N	21090
N (participant)	555
N (behavior)	38
Prediction accuracy	0.83
AUC	0.91

All continuous predictors are mean-centered and scaled by 1 standard deviation. Coefficient estimates are odds ratios. Numbers in the brackets represent 95% confidence intervals.

a p < 0.001.

b p < 0.01.

Finally, given the literature on how individual level factors such as climate beliefs, risk perception and emotional reactions influence pro-environmental behavior, we have formulated an additional model, in which the likelihood of adoption was predicted by individual level variables only (see Model 2 in Table.S3). On average, individuals with higher risk perceptions (OR = 1.16, 95%CI = [1.05, 1.28], p = 0.003), stronger efficacy beliefs (OR = 1.2, 95%CI = [1.11, 1.31], p < 0.001), and stronger positive emotional engagement with climate change (OR = 1.14, 95%CI = [1.06, 1.22], p < 0.001) were more likely to adopt mitigative behaviors. These results suggests that even though emotional reactions to climate change, risk perceptions, and efficacy beliefs are associated with pro-environmental behavior, these individual level factors did not predict the adoption of mitigative behaviors beyond the perceptions of different behaviors. This finding further underlines the importance of studying within-person affective and perceptual differences that are related to various behaviors to understand the adoption of impactful sustainable behavior.

## Discussion

We assessed the relationship between the perceived and actual impact of mitigative human behaviors. Our results give a clear indication that the mitigation potential of various human behaviors and their perceived impact are unrelated, highlighting that people may neglect to appropriately consider the effectiveness of their behaviors when deciding what action to take. Instead, our results suggest that perceived impact are grounded in affective experiences. We find that people perceive high-impact behaviors to be more negative and harder to adopt. Consequently, having positive affective reactions to a behavior is related to having a higher impact perception. Thus, low-impact behaviors are perceived as more positive and easier to complete and are adopted more frequently. Further, having higher emotional engagement with climate change is associated with a higher impact perception in general. In addition, having a positive affective association with a behavior is a robust predictor for the adoption of that behavior beyond its actual mitigation potential as well as its perceived impact and difficulty. Although our data does not permit a direct causal explanation, the results provide clear evidence that (1) people have a rather poor understanding of the relative impact of human behaviors in reducing carbon emissions, and (2) affect and emotions play an essential role in explaining impact judgments and which behaviors are more frequently adopted. Hence, our findings complement the literature suggesting the existence of an impact neglect in valuation judgments of sustainable behaviors.^14^^,^^15^^,^^16^^,^^18^^,^^19^ We also extend the current understanding by showing how affective associations with mitigative behaviors from various consumption domains are related to perceived impact and adoption of these behaviors.

The current findings show important differences in people’s perceptions of high- and low-impact behaviors. People like and undertake behaviors with relatively low impact and think that high-impact behaviors are difficult to complete. How can we interpret these differences? People act in a way, so they feel that they make a personal difference^37^; and their actions are meaningful.^38^ Thus, the issue at hand may not be the lack of intention or motivation, but rather what makes people feel that they made a difference. We need to feel that our actions have an impact; and knowing that our actions will have an impact in a non-proximate future may not generate the needed affective response.^39^^,^^40^^,^^41^ The impact of effective pro-environmental action is inherently a very abstract phenomenon (e.g., isolating a house), that only makes you rely on factual knowledge and not experiential. Furthermore, high-impact behaviors often imply that you must give something up (e.g., not flying abroad for a vacation, not eating meat), not leaving you with the feeling of making a personal difference, but instead with negative affective associations. This lack of positive affective experience may decrease perceived impact, at the same time fueling perceived difficulty and psychological barriers in front of a high-impact behavior. On the other hand, low-impact behaviors that are easy to complete may induce a sense of accomplishment, which may enhance positive affect. We should stress that the correlational nature of our study does not permit a direct causal explanation between affective associations, perceived impact, and behavior. For instance, a belief that a certain behavior has a high impact could lead to a positive affective reaction to it. However, it could also be the other way around: a positive reaction induced by a behavior could lead to an increased impact perception. The affect heuristic perspective would suggest that the causal link between impact judgment and affect could be a two-way reinforcing effect.^26^ However, more directed experimental research is needed to uncover the causal mechanism, which would potentially be very useful to inform interventions aimed at motivating high-impact sustainable behaviors.

The current findings have potential policy implications for how we can achieve impactful pro-environmental behavior. Behavioral research has studied information-based interventions, aiming to increase impact knowledge, which is very important. However, research within the broader pro-social domain highlights the risk with interventions aiming to increase impactful behavior by redirecting the focus to the outcome and at the same time ignoring the role of affective processes in motivation.^42^ Thus, using information aimed at decreasing the influence of affect and emotion might reduce overall pro-environmental behavior. Suppose an individual believes that a certain behavior they adopt has a considerable impact on reducing emissions. In this case, information to convince them otherwise may fail or even backfire as this may decrease the positive affect the person experiences and demotivate them to engage in future pro-environmental behavior. Hence, we argue for a new line of interventions that focus on the motivational energy in effect and emotion, contrary to interventions aiming to reduce the negative impact of emotions by increasing more logical and deliberative judgments. This kind of affect-focused interventions should ideally strive for enhancing positive affect associated with high-impact behaviors with the aim of reinforcing an impactful pro-environmental behavioral pattern by direct motivational processes, but also by increasing the perceived impact and/or decreasing perceived difficulty. Future research should study how to harness the potential of this type of positive affective feedback loop to motivate impactful mitigative behaviors.^2^^,^^11^^,^^43^^,^^44^ More focused research on the causes of affective reactions to high-impact behaviors could guide the creation of affect-focused interventions. However, concerns could be raised regarding if this would increase impact beliefs in general, that in turn could create a type of optimism bias, decreasing the perception of risk associated with climate change and demotivating behavior.^45^^,^^46^^,^^47^ Contrary to this, we suggest that focusing on the low-impact behaviors, which are often highlighted in public policy and campaigns, reinforces the view that whatever you do for the environment is better than nothing, a view that already fuels this environmental optimism bias. Instead, affect-focused interventions can help dispel the optimistic allure of feeling that you have done your part when completing a low-impact behavior.

### Limitations of the study

The current study, albeit presenting clear evidence for the relationship between affective reactions, impact judgments, and adoption of mitigative behaviors, is correlational. Thus, we cannot definitively show how the causal links between these factors look like. We could, however, speculate based on affect heuristic research^26^ showing that easily available mental tags induced by affective reactions enhance subsequent judgments about the benefits of a certain behavior. However, it is important to definitively provide evidence for this causal relationship and to study the underlying causes of people’s affective reactions to mitigative behaviors. We believe the current study by evidencing the robust relationship between affect, impact judgments, and pro-environmental behavior could be a starting point for asking more directed questions to uncover the responsible mechanisms.

The participants in this study were recruited online from a US sample. We think this sample, albeit not representing the general population, may be relevant for the aims of this specific study because the human populations that need to adjust their behavior the most are from large emitter countries in the Global North. However, we still think it is worthwhile for future studies to collect data from diverse populations, as public perceptions of and emotional reactions to sustainability issues may differ between different cultures.^11^

### Conclusions

The current study provides clear evidence that affective processes play an important role in perceived impact and adoption of various mitigative behaviors. However, to reach the goal of mobilizing people to change their behavioral patterns to reduce emissions, we need to improve our understanding of the mechanisms through which affect and emotions influence judgments and decision-making in the sustainability domain. Experienced affect and emotion in the here and now can motivate behavior by amplifying our needs^28^ and signaling whether our values are supported or threatened,^48^ anticipated emotions can incentivize current action,^49^^,^^50^^,^^51^ and emotions help us gauge overall goal attainment.^52^^,^^53^ But the role of affect in judgment and decision-making has not been extensively studied in the context of sustainable behaviors.^2^ Therefore, it is of utmost importance for future research to fully understand how affect and emotions influence impact neglect and sustainable behavior, to be able to better mobilize people with interventions aimed to enhancing their positive affective associations.

## STAR★Methods

### Key resources table

**Table:** 

REAGENT or RESOURCE	SOURCE	IDENTIFIER
**Deposited data**		

Survey data	This paper	OSF: https://osf.io/e6ncy/

**Software and algorithms**		

R Project for statistical computing	http://www.r-project.org/	RRID:SCR_001905
R package: lme4	https://cran.r-project.org/web/packages/lme4/index.html	RRID:SCR_015654
R package: lmerTest	http://CRAN.R-project.org/package=lmerTest	RRID:SCR_015656
R package: tidyverse	https://CRAN.R-project.org/package=tidyverse	RRID:SCR_019186
Qualtrics Survey Platform	https://www.qualtrics.com/research-core/survey-software/	RRID:SCR_016728

### Resource availability

#### Lead contact

Further information and requests for resources and reagents should be directed to and will be fulfilled by the lead contact, Erkin Asutay (erkin.asutay@liu.se).

#### Materials availability

This study did not generate new unique reagents.

### Experimental model and study participant details

#### Participants

The participants were recruited online (Prolific) from a US sample and responded through an online survey tool (Qualtrics Survey Platform). We consider a US sample relevant for the current research aims, since the human population that needs to change their lifestyle and consumer choices the most is the citizens in the Global North and large emitter countries, which is led by the USA. Therefore, we investigated impact perceptions, affective reactions, and pro-environmental behavior in a US sample.

Informed consent was collected from all participants. The study was deemed exempt from regular ethical approval since all data is anonymous. Data from a total of 600 participants were collected. To assure the quality of responses we have included attention check questions (four questions distributed in the survey) that require participants to read the prompts carefully and select a predetermined response. Further, we have studied the distribution of individuals’ responses within each block and removed participants with low-quality data. We removed 34 participants for providing the same response to all questions within at least one survey block, 10 individuals for failing at least one attention check question, and one participant for attending twice. Hence, we removed 45 individuals (7.5% of the total sample) prior to any analyses due to low quality and/or inattentive responding. The final sample consisted of 555 participants after exclusions (272 male, 281 female, 1 non-binary, 1 did not disclose; mean-age = 38.6, sd-age = 13.4; see Table.S1 for sample characteristics).

### Method details

#### Selection of mitigative behaviors

We selected the mitigative behaviors from a recent review on quantifying the mitigation potentials of various human behaviors in terms of estimated reduced carbon emissions per capita per year.^36^ In the original article, Ivanova and colleagues systematically review the literature on mitigation potentials across various consumption domains, including food, transport, and housing. We first excluded behaviors with less than 4 studies in the original meta-analysis.^36^ Then, we examined the original sources for each behavior closely and combined categories when it was warranted (i.e., similar behaviors with overlapping ranges of estimated mitigation potentials). For instance, ‘*purchasing fewer appliances’*, ‘*purchasing fewer and durable items’*, and ‘*purchasing less textiles’* were combined into ‘*purchasing fewer and more durable things (appliances, furniture, textiles).’* We calculated new means for the estimated mitigation potentials when we combined behaviors based on the accompanying raw data from Ivanova et al.^36^ The final list consisted of 38 human behaviors (Table.S2).

#### Subjective ratings for mitigative behaviors

*Perceived impact.* To assess the individuals’ impact perception related to various mitigative behaviors, we ask participants to rate the impact of each behavior to reduce carbon emissions if it is adopted by the average citizen on a scale from 1 (very small impact) to 5 (very large impact) in a randomized order.

*Affective reactions.* In a separate block, participants reported how they feel when they think of each behavior (*“Thinking about this behavior I feel …”*) on a five-point scale (1 = very negative, 5 = very positive). This question was aimed to assess affective reactions to mitigative behaviors. Here, we used a bipolar scale, so a lower score represents an existence of a negative affective reaction, while a higher score reflects a positive affective reaction.

*Perceived difficulty.* We also assessed, in a separate block, how difficult individuals perceive to adopt mitigative behaviors. Participants responded to the question, *“How difficult would it be for you to adopt the following behaviors in your current living situation?”* on a scale ranging from 1 (very easy) to 5 (very difficult).

*Current pro-environmental behavior.* Finally, participants viewed the complete list of mitigative behaviors and clicked on those they are currently undertaking (for continued behaviors such as *consuming organic food*) or have already done (for one-time behaviors such as *improving thermal insulation of my home*). This section of the survey was used to assess current sustainable behavior of individuals as well as the adoption rate for the behaviors.

The order of the first two blocks (i.e., perceived impact and affective reactions) were balanced among participants. We have looked for possible differences due to different block orders. Participants who completed the affect block before the perceived impact block reported more positive affective reactions (t(551) = 4.6, p < 0.001) and higher perceived impact (t(544) = 5.6, p < 0.001) in comparison to those completed the perceived impact block first (Table.S4). Apart from this, we found no other differences due to the block order (see Figure S3).

#### Control questions

After the questions about various mitigative behaviors, we ask participants to respond to control questions, which assessed their belief in the human-caused climate change, belief that collective action can reduce climate change as well as general risk perception for and emotional reactions to climate change.

*Belief in the human-caused climate change.* The individuals rated to what degree they agreed with three statements on a scale from one (strongly disagree) to five (strongly agree). The statements were, *“I am certain that climate change is really happening”*, *“I am certain that the human activity is the main cause of climate change”*, and *“I believe that doing something to reduce climate change is important.”* A mean score was calculated, in which a higher score reflects a stronger belief in the human-caused climate change (Cronbach’s alpha = 0.8).

*Risk perception*. Participants rated how much risk the climate change poses to self (two items), to others (two items), and to non-human populations of earth (two items) on a visual analogue scale (VAS scale) ranging from 0 (Climate change does not present any risk) to 100 (Climate change presents an extreme risk). An aggregate measure of perceived risk was calculated (Cronbach’s alpha = 0.93).

*Belief in outcome efficacy.* To measure outcome efficacy beliefs, participants rated to what degree they agreed with three statements on a scale from one (strongly disagree) to five (strongly agree). The statements were, *“There is no urgency about taking individual action on climate change because new technologies will be developed to solve the issue of climate change”*, *“I believe we can act collectively and make a difference in reducing climate change”*, and *“The behavior individuals engage in to reduce carbon emissions will make no real difference in reducing climate change.”* The first and third statements were reverse coded and an aggregate measure of belief about outcome efficacy was calculated (Cronbach’s alpha = 0.68). A high score on the scale means that the participant has a strong belief that actions and behaviors of individuals will help reduce climate change.

*Emotional reactions to climate change*. Participants reported the extent they felt a list of 11 emotions when they think of climate change on a scale ranging from one (not at all) to five (extremely). Positive emotions included determined, hopeful, inspired, proud, and strong (Cronbach’s alpha = 0.75). Negative emotions consisted of afraid, anxious, ashamed, distressed, guilty, and worried (Cronbach’s alpha = 0.9). We selected these emotions based on the accumulating research on emotional experiences induced by climate crisis as well as emotional reactions to climate communications.^2^^,^^5^^,^^9^^,^^54^ We do not claim that this is a definitive list of emotional states related to sustainability issues. However, these emotional states have been shown to be relevant to climate change. We calculated composite positive and negative emotional reactions to climate change.

### Quantification and statistical analysis

#### The relationship between the subjective impact ratings and the objective mitigation potentials

First, we investigated the relationship between subjective perception (i.e., perceived impact, affective reactions, and perceived difficulty), adoption rate, and the estimated mitigation potential of the behaviors. We calculated, for each behavior, the average perceived impact, affective reaction, and difficulty as well as the proportion of participants adopting the behavior. Then, we calculated Spearman’s rank correlations between the average ratings, adoption rate, and the mitigation potentials. This analysis reveals whether average ratings and adoption rate for mitigative behaviors correlate with their objective impact in terms of estimated reduction of carbon emissions.

Moreover, to further study whether impact judgments reflect objective mitigative impact in reducing carbon emissions, we quantified the relationship between perceived and objective impact separately for each individual using Spearman rank correlation. This analysis yields one correlation coefficient (ranging from −1 to 1) per participant reflecting whether the individual’s impact perceptions are in line with the objective impact. We studied the distribution of this statistic (i.e., the proportion of participants having impact judgments positively correlated with estimated mitigation potentials).

#### Determinants of impact judgments

We studied the predictors of perceived impact using mixed-effects linear regression models. By-individual and by-behavior random intercepts were included in the models to account for random variations between individuals in responding and variations due to the selected behaviors. Random slopes were fitted unless the inclusion of them resulted in convergence issues or did not improve model fits compared to a fixed-effects-plus-random-intercept model indicated by likelihood ratio tests. The modeling of perceived impact was done through the following steps. We first included behavior-level fixed effects: perceived difficulty, positive affect, and the estimated mitigation potential (i.e., the objective impact). Next, we included positive and negative emotional reactions to climate change at the individual level. Finally, we included other control variables in the model, i.e., risk perceptions and beliefs in human-caused climate change and outcome efficacy. This analysis reveals the determinants of perceived impact related to both the behaviors and the participants. These models help us determine how affective reactions and perceived difficulty are associated with impact perception while controlling for the objective impact of behaviors. Further, we could study how individual differences in emotional reactions to climate change, risk perception, and climate beliefs influence perceived impact of mitigative behaviors.

#### Determinants of current pro-environmental behavior

We studied the predictors for the likelihood of adopting a mitigative behavior using mixed-effects logistic regression models. The dependent variable was whether the participants adopt the behaviors. The rest of the analysis strategy was the same as in perceived impact analysis explained above. We first formulated a model with behavior-level predictors: perceived impact, affective reactions, perceived difficulty, and estimated mitigation potentials. We then included positive and negative emotions, and finally, other control variables (risk perception and climate beliefs).

## Data Availability

• The dataset to perform the statistical analysis is deposited on OSF (https://osf.io) and is publicly available as of the date of publication (https://osf.io/e6ncy/). • The code used for statistical analysis is deposited on OSF (https://osf.io) and is publicly available as of the date of publication (https://osf.io/e6ncy/). • The original survey questions are deposited on OSF (https://osf.io) and are publicly available as of the date of publication (https://osf.io/e6ncy/).

## Supplemental information

Document S1. Supplemental results, Figures S1–S2, and Tables S1–S4

## References

[1] IPCC (2022).

[2] Brosch T., Steg L. (2021). Leveraging emotion for sustainable action. One Earth.

[3] Dietz T., Gardner G.T., Gilligan J., Stern P.C., Vandenbergh M.P. (2009). Household actions can provide a behavioral wedge to rapidly reduce US carbon emissions. Proc. Natl. Acad. Sci. USA.

[4] Nielsen K.S., Clayton S., Stern P.C., Dietz T., Capstick S., Whitmarsh L. (2021). How psychology can help limit climate change. Am. Psychol..

[5] Steg L. (2023). Psychology of climate change. Annu. Rev. Psychol..

[6] Whitmarsh L., Poortinga W., Capstick S. (2021). Behaviour change to address climate change. Curr. Opin. Psychol..

[7] Hampton S., Whitmarsh L. (2023). Choices for climate action: A review of the multiple roles individuals play. One Earth.

[8] Nielsen K.S., Cologna V., Lange F., Brick C., Stern P.C. (2021). The case for impact-focused environmental psychology. J. Environ. Psychol..

[9] Brosch T. (2021). Affect and emotions as drivers of climate change perception and action: a review. Current Opinion in Behavioral Sciences.

[10] Pessoa L. (2009). How do emotion and motivation direct executive control?. Trends Cogn. Sci..

[11] Schneider C.R., van der Linden S. (2023). An emotional road to sustainability: How affective science can support pro-climate action. Emotion Review.

[12] Capstick S., Whitmarsh L., Poortinga W., Pidgeon N., Upham P. (2015). International trends in public perceptions of climate change over the past quarter century. WIREs Climate Change.

[13] Steg L. (2018). Limiting climate change requires research on climate action. Nat. Clim. Chang..

[14] Wynes S., Nicholas K.A. (2017). The climate mitigation gap: education and government recommendations miss the most effective individual actions. Environ. Res. Lett..

[15] Attari S.Z., DeKay M.L., Davidson C.I., Bruine de Bruin W. (2010). Public perceptions of energy consumption and savings. Proc. Natl. Acad. Sci. USA.

[16] Camilleri A.R., Larrick R.P., Hossain S., Patino-Echeverri D. (2019). Consumers underestimate the emissions associated with food but are aided by label. Nat. Clim. Chang..

[17] Cologna V., Berthold A., Siegrist M. (2022). Knowledge, perceived potential and trust as determinants of low- and high-impact pro-environmental behaviours. J Env Psych.

[18] Holmgren M., Andersson H., Sörqvist P. (2018). Averaging bias in environmental impact estimates: Evidence from the negative footprint illusion. J. Environ. Psychol..

[19] Wynes S., Zhao J., Donner S.D. (2020). How well do people understand the climate impact of individual actions?. Climatic Change.

[20] Kok A.L., Barendregt W. (2021). Why Ecological Footprint Calculators Should Move beyond Information Provision – An Empirical Study of the Relationship between Environmental Knowledge and Ecological Footprint. Preprints.

[21] Staats H.J., Wit A.P., Midden C.Y.H. (1996). Communicating the greenhouse effect to the public: evaluation of a mass media campaign from a social dilemma perspective. J. Environ. Manag..

[22] Steg L. (2012).

[23] George J.M., Dane E. (2016). Affect, emotion, and decision making. Organ. Behav. Hum. Decis. Process..

[24] Phelps E.A., Lempert K.M., Sokol-Hessner P. (2014). Emotion and decision-making: Multiple modulatory neural circuits. Annu. Rev. Neurosci..

[25] Lerner J.S., Li Y., Valdesolo P., Kassam K.S. (2015). Emotion and decision-making. Annu. Rev. Psychol..

[26] Slovic P., Finucane M.L., Peters E., MacGregor D.G. (2007). The affect heuristic. Eur. J. Oper. Res..

[27] Weber E.U., Johnson E.J. (2009). Mindful judgment and decision-making. Annu. Rev. Psychol..

[28] Clore G.L., Huntsinger J.R. (2007). How emotions inform judgment and regulate thought. Trends Cogn. Sci..

[29] Schwarz N. (2000). Emotion, cognition, and decision making. Cogn. Emot..

[30] Jia L., van der Linden S. (2020). Green but not altruistic warm-glow predicts conservation behavior. Conserv. Sci. Pract..

[31] Taufik D., Bolderdijk J.W., Steg L. (2016). Going green? The relative importance of feelings over calculation in driving environmental intent in The Netherlands and the United States. Energy Res. Soc. Sci..

[32] Odou P., Schill M. (2020). How anticipated emotions shape behavioral intentions to fight climate change. J. Bus. Res..

[33] Geiger J.L., Steg L., van der Werff E., Ünal A.B. (2019). A meta-analysis of factors related to recycling. J. Environ. Psychol..

[34] Hartmann P., Eisend M., Apaolaza V., D’Souza C. (2017). Warm glow vs. altruistic values: How important is intrinsic emotional reward in proenvironmental behavior?. J. Environ. Psychol..

[35] Van Der Linden S. (2018). Warm glow is associated with low- but not high-cost sustainable behaviour. Nat. Sustain..

[36] Ivanova D., Barrett J., Wiedenhofer D., Macura B., Callaghan M., Creutzig F. (2020). Quantifying the potential for climate change mitigation of consumption options. Environ. Res. Lett..

[37] Duncan B. (2004). A theory of impact philanthropy. J. Publ. Econ..

[38] Venhoeven L.A., Bolderdijk J.W., Steg L. (2020). Why going green feels good. J. Environ. Psychol..

[39] Chang H.H., Pham M.T. (2018). Affective Boundaries of Scope Insensitivity. J. Consum. Res..

[40] Chang H.H., Tsai P.H., Liu C.W., Ku H.C., Kao C.C., Kao Y.H. (2013). Affect as a Decision-Making System of the Present. Toxicol. Lett..

[41] Seabright M.A. (2010). The role of the affect heuristic in moral reactions to climate change. J. Global Ethics.

[42] Small D.A., Loewenstein G., Slovic P. (2007). Sympathy and callousness: The impact of deliberate thought on donations to identifiable and statistical victims. Organ. Behav. Hum. Decis. Process..

[43] Fredrickson B.L., Joiner T. (2018). Reflections on positive emotions and upward spirals. Perspect. Psychol. Sci..

[44] Van Cappellen P., Rice E.L., Catalino L.I., Fredrickson B.L. (2018). Positive affective processes underlie positive health behaviour change. Psychol. Health.

[45] Gifford R. (2011). The dragons of inaction: psychological barriers that limit climate change mitigation and adaptation. Am. Psychol..

[46] Hatfield J., Soames Job R. (2001). Optimism bias about environmental degradation: The role of the range of impact of precautions. J. Environ. Psychol..

[47] Pahl S., Harris P.R., Todd H.A., Rutter D.R. (2005). Comparative optimism for environmental risks. J. Environ. Psychol..

[48] Perlaviciute G., Steg L., Contzen N., Roeser S., Huijts N. (2018). Emotional responses to energy projects: insights for responsible decision making in a sustainable energy transition. Sustainability.

[49] Mellers B.A., McGraw A.P. (2001). Anticipated emotions as guides to choice. Curr. Dir. Psychol. Sci..

[50] Schneider C.R., Zaval L., Weber E.U., Markowitz E.M. (2017). The influence of anticipated pride and guilt on pro-environmental decision making. PLoS One.

[51] Van Der Schalk J., Bruder M., Manstead A. (2012). Regulating emotion in the context of interpersonal decisions: the role of anticipated pride and regret. Front. Psychol..

[52] Carver C.S. (2015). Control processes, priority management, and affective dynamics. Emotion Review.

[53] Carver C.S., Scheier M.F., Robinson M.D., Watkins E.R., Harmon-Jones E. (2013). Guilford Handbook of Cognition and Emotion.

[54] Schneider C.R., Zaval L., Markowitz E.M. (2021). Positive emotions and climate change. Curr. Opin. Behav. Sci..

