# Supplementary material for: Affective responses drive the impact neglect in sustainable behavior
Source: iScience. 2023 Oct 20;26(11):108280. doi: 10.1016/j.isci.2023.108280 (PMC10661116; doi:10.1016/j.isci.2023.108280)
Supplement: Document S1. Supplemental results, Figures S1–S2, and Tables S1–S4 [file mmc1.pdf]

**iScience, Volume *26***

## Supplemental information

# Affective responses drive the impact neglect in sustainable behavior

**Erkin Asutay, Hulda Karlsson, and Daniel Västfjäll**

# SUPPLEMENTARY RESULTS

**Table S1.** Sample characteristics, related to STAR Methods.

| | |
|---|---|
| N | 555 |
| Age | 38.61 (13.36) |
| Gender | |
| Men | 272 (49 %) |
| Women | 281 (50.6 %) |
| Non-binary | 1 (0.2 %) |
| Did not disclose | 1 (0.2 %) |
| Income | |
| < 24,999 $ | 20.9 % |
| 25,000 – 49,999 $ | 29.0 % |
| 50,000 – 74,999 $ | 19.1 % |
| 75,000 – 99,999 $ | 15.3 % |
| 100,001 – 124,999 $ | 8.1 % |
| > 125,001 $ | 7.6 % |
| Education | |
| Grades 1-11 | 1.1 % |
| Graduated from high school | 31.7 % |
| Graduated from college | 38.9 % |
| Completed graduate school | 28.3 % |
| | |
| Belief in human-caused climate change | 4.4 (0.7) |
| Belief in response efficacy | 3.9 (0.8) |
| Positive climate emotions | 2.4 (0.7) |
| Negative climate emotions | 3.0 (1.0) |
| Risk perception | 73 (21) |

Belief in human-caused climate change, belief in response efficacy, positive and negative climate emotions have a range from 1 to 5. Risk perception scale extends from 0 to 100.

**Table S2.** Descriptive statistics for mitigative behaviors, related to STAR Methods

| Behavior | Average mitigation potential [tCO2e/cap/year] | Adoption (%) | Perceived impact | (sd) | Affect | (sd) | Difficulty | (sd) |
|---|---|---|---|---|---|---|---|---|
| Living car-free | 2.10 | 0.18 | 3.94 | 1.10 | 2.69 | 1.33 | 3.83 | 1.39 |
| Shifting to BEV | 2.01 | 0.04 | 3.32 | 1.10 | 3.36 | 1.21 | 3.52 | 1.25 |
| One less flight (long return) | 1.89 | 0.41 | 3.32 | 1.18 | 3.57 | 1.14 | 2.06 | 1.22 |
| Shift to renewable electricity | 1.46 | 0.20 | 4.15 | 0.89 | 4.26 | 0.90 | 2.95 | 1.26 |
| Using public transport | 0.99 | 0.31 | 3.59 | 0.99 | 3.16 | 1.25 | 3.07 | 1.40 |
| Eating a vegan diet | 0.91 | 0.10 | 2.94 | 1.22 | 2.37 | 1.30 | 3.68 | 1.31 |
| Reduce air transport | 0.83 | 0.44 | 3.97 | 1.02 | 3.63 | 1.11 | 2.28 | 1.21 |
| Walking and/or biking more | 0.79 | 0.69 | 3.78 | 1.04 | 4.12 | 0.92 | 2.22 | 1.17 |
| Having no pets | 0.78 | 0.38 | 1.95 | 1.00 | 2.23 | 1.37 | 3.13 | 1.70 |
| Driving less | 0.77 | 0.46 | 3.79 | 0.97 | 3.59 | 1.09 | 3.04 | 1.31 |
| Installing a heat pump | 0.75 | 0.03 | 2.98 | 1.03 | 3.14 | 1.06 | 3.63 | 1.12 |
| Shifting to a (P)HEV | 0.73 | 0.04 | 3.29 | 1.09 | 3.36 | 1.18 | 3.60 | 1.20 |
| One less flight (medium return) | 0.62 | 0.38 | 3.16 | 1.17 | 3.56 | 1.16 | 2.07 | 1.16 |
| Adopting a Mediterranean diet | 0.59 | 0.31 | 3.26 | 1.13 | 3.35 | 1.21 | 2.62 | 1.22 |
| Improved cooking equipment | 0.55 | 0.35 | 2.90 | 1.06 | 3.79 | 0.94 | 2.75 | 1.14 |
| Increase product and service sharing | 0.52 | 0.19 | 3.16 | 1.03 | 3.42 | 0.98 | 2.89 | 1.02 |
| Adopting a vegetarian diet | 0.52 | 0.17 | 3.00 | 1.16 | 2.85 | 1.36 | 3.09 | 1.36 |
| Partially shifting to low-carbon meats | 0.48 | 0.29 | 2.66 | 1.10 | 3.23 | 1.19 | 2.30 | 1.16 |

| Behavior | Average mitigation potential [tCO2e/cap/year] | Adoption (%) | Perceived impact | (sd) | Affect | (sd) | Difficulty | (sd) |
|---|---|---|---|---|---|---|---|---|
| Eating organic food | 0.47 | 0.24 | 2.66 | 1.09 | 3.30 | 1.08 | 2.76 | 1.11 |
| Telecommuting | 0.45 | 0.28 | 3.26 | 1.08 | 3.60 | 1.03 | 2.58 | 1.24 |
| Eating local-produced food | 0.44 | 0.39 | 3.53 | 1.06 | 4.13 | 0.90 | 2.42 | 0.99 |
| Shifting to a smaller car | 0.42 | 0.17 | 3.00 | 1.08 | 3.29 | 1.13 | 2.95 | 1.28 |
| Reduce living space | 0.34 | 0.10 | 2.56 | 1.03 | 2.56 | 1.07 | 3.80 | 1.09 |
| Carpooling | 0.32 | 0.13 | 3.35 | 1.02 | 3.18 | 1.16 | 3.33 | 1.25 |
| Reduce food waste | 0.32 | 0.85 | 3.62 | 1.06 | 4.45 | 0.76 | 1.78 | 0.88 |
| Consuming energy and material efficient goods | 0.29 | 0.34 | 3.63 | 0.94 | 4.01 | 0.82 | 2.51 | 0.91 |
| Installing smart thermostat | 0.23 | 0.24 | 3.08 | 1.06 | 3.81 | 1.03 | 2.56 | 1.18 |
| Thermal insulation | 0.18 | 0.28 | 3.89 | 0.97 | 4.31 | 0.81 | 3.12 | 1.22 |
| Purchase product with less and/or greener packaging | 0.16 | 0.51 | 3.56 | 1.05 | 4.21 | 0.89 | 2.24 | 0.95 |
| Reduce indoor temperature by 1-3 degrees | 0.14 | 0.59 | 3.08 | 1.06 | 3.48 | 1.05 | 2.27 | 1.14 |
| Using energy efficient home appliances | 0.11 | 0.53 | 3.57 | 1.03 | 4.27 | 0.78 | 2.31 | 1.00 |
| Consuming less processed food | 0.10 | 0.55 | 2.77 | 1.06 | 3.52 | 1.07 | 2.49 | 1.11 |
| Purchasing fewer and more durable goods | 0.08 | 0.65 | 3.66 | 1.01 | 4.20 | 0.87 | 2.20 | 0.97 |
| Less plastic | 0.08 | 0.71 | 3.87 | 1.05 | 4.36 | 0.79 | 2.38 | 1.08 |
| Improve energy efficiency in laundry | 0.07 | 0.75 | 3.33 | 1.07 | 3.97 | 0.94 | 2.08 | 1.03 |

| Behavior | Average mitigation potential [tCO2e/cap/year] | Adoption (%) | Perceived impact | (sd) | Affect | (sd) | Difficulty | (sd) |
|---|---|---|---|---|---|---|---|---|
| Recycling | 0.06 | 0.91 | 3.81 | 1.08 | 4.50 | 0.78 | 1.54 | 0.85 |
| Composting | 0.03 | 0.37 | 3.10 | 1.15 | 3.85 | 1.05 | 2.29 | 1.21 |
| Using less paper | 0.01 | 0.66 | 3.12 | 1.15 | 3.95 | 0.96 | 2.06 | 1.02 |

**Table S3.** Logistic mixed models predicting the individuals' likelihood of undertaking a mitigative behavior based on behavior-level predictors (Model 1) and individual-level factors (Model 2). The individual level factors did not reliably contribute to the model (i.e., non-significant estimates, unaffected performance metric, unchanged behavior-level estimates). Related to Table 2.

| | Model 1 | Model 2 | Model 3 |
|---|---|---|---|
| Positive affect | **2.15 [1.99, 2.32] *** ** | | **2.15 [1.99, 2.32] *** ** |
| Perceived difficulty | **0.32 [0.30, 0.35] *** ** | | **0.32 [0.30, 0.35] *** ** |
| Perceived impact | **1.28 [1.20, 1.36] *** ** | | **1.28 [1.20, 1.36] *** ** |
| Mitigation potential | **0.58 [0.41, 0.82] ** ** | | **0.58 [0.41, 0.82] ** ** |
| Positive climate emotions | | **1.14 [1.06, 1.22] *** ** | 1.04 [0.96, 1.12] |
| Negative climate emotions | | 1.07 [0.98, 1.16] | 1.03 [0.94, 1.13] |
| Risk perception | | **1.16 [1.05, 1.28] ** ** | 1.10 [0.99, 1.23] |
| Belief in climate change | | 0.9 [0.79, 0.99] | 0.9 [0.79, 0.99] |
| Belief in outcome efficacy | | **1.21 [1.11, 1.31] *** ** | 1.02 [0.93, 1.12] |
| (Intercept) | **0.34 [0.24, 0.48] *** ** | **0.48 [0.31, 0.74] *** ** | **0.34 [0.24, 0.48] *** ** |
| N | 21090 | 21090 | 21090 |
| N (pid) | 555 | 555 | 555 |
| N (action) | 38 | 38 | 38 |
| Prediction accuracy | 0.83 | 0.76 | 0.83 |
| AUC | 0.91 | 0.82 | 0.91 |

All continuous predictors are mean-centered and scaled by 1 standard deviation. Coefficient estimates are odds ratios. Numbers in the brackets represent 95% confidence intervals. *** p < 0.001; ** p < 0.01; * p < 0.05.

**Table S4.** Affect and impact rating differences based on block orders, Related to STAR Methods.

| **Variable** | **Block Order** | **N** | **Mean (SD)** | **Two sample t-test** | | |
|---|---|---|---|---|---|---|
| | | | | t | df | p |
| Affect | Affect first | 268 | 3.71 (0.54) | 4.63 | 551 | <.001 |
| | Impact first | 287 | 3.49 (0.54) | | | |
| Impact | Affect first | 268 | 3.46 (0.66) | 5.6 | 544 | <.001 |
| | Impact first | 287 | 3.16 (0.63) | | | |

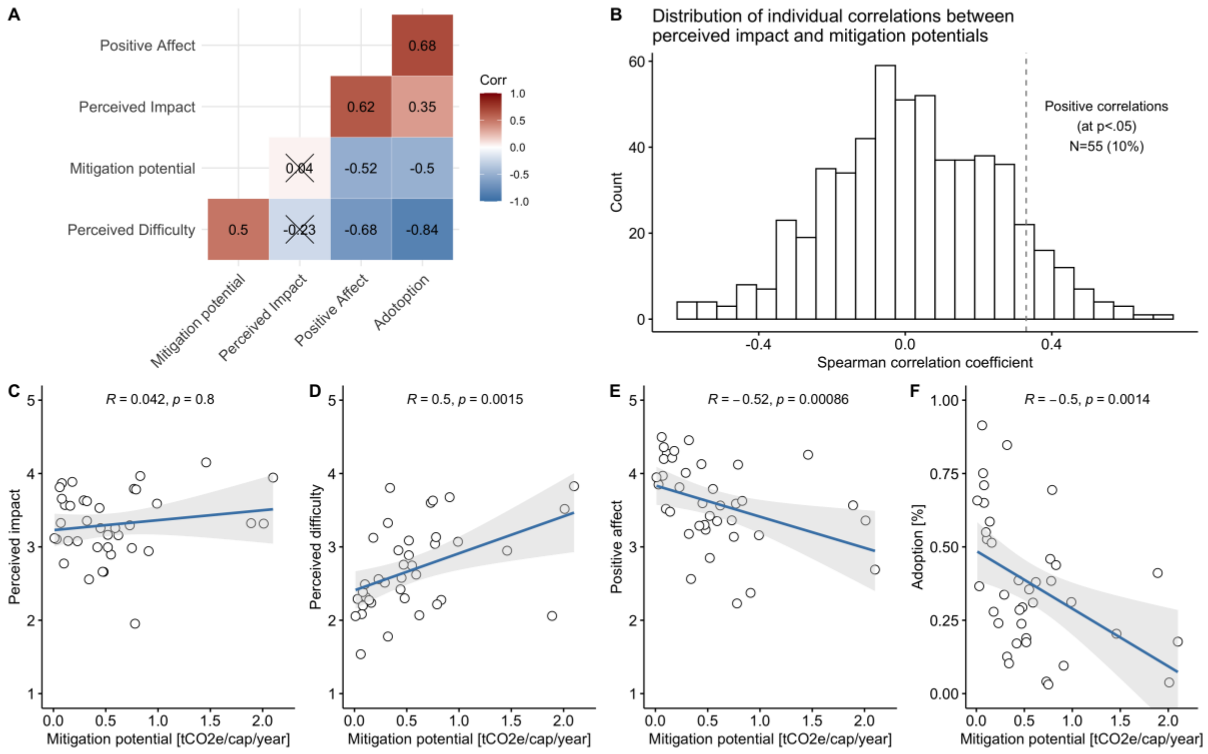


**Figure S1.** Spearman correlations between perception of mitigative behaviors, estimated mitigation potentials, and adoption rate (Panel A). The scatterplots that show the relationship between responses and the estimated mitigation potentials are presented in panels C, D, E, and F (presented statistics are the same as in Panel A). The distribution of individual correlations between impact judgments and the estimated mitigation potentials are presented in Panel B. Impact judgments were positively associated with the estimated mitigation potentials for 10 % of the participants. Related to Figure 1.

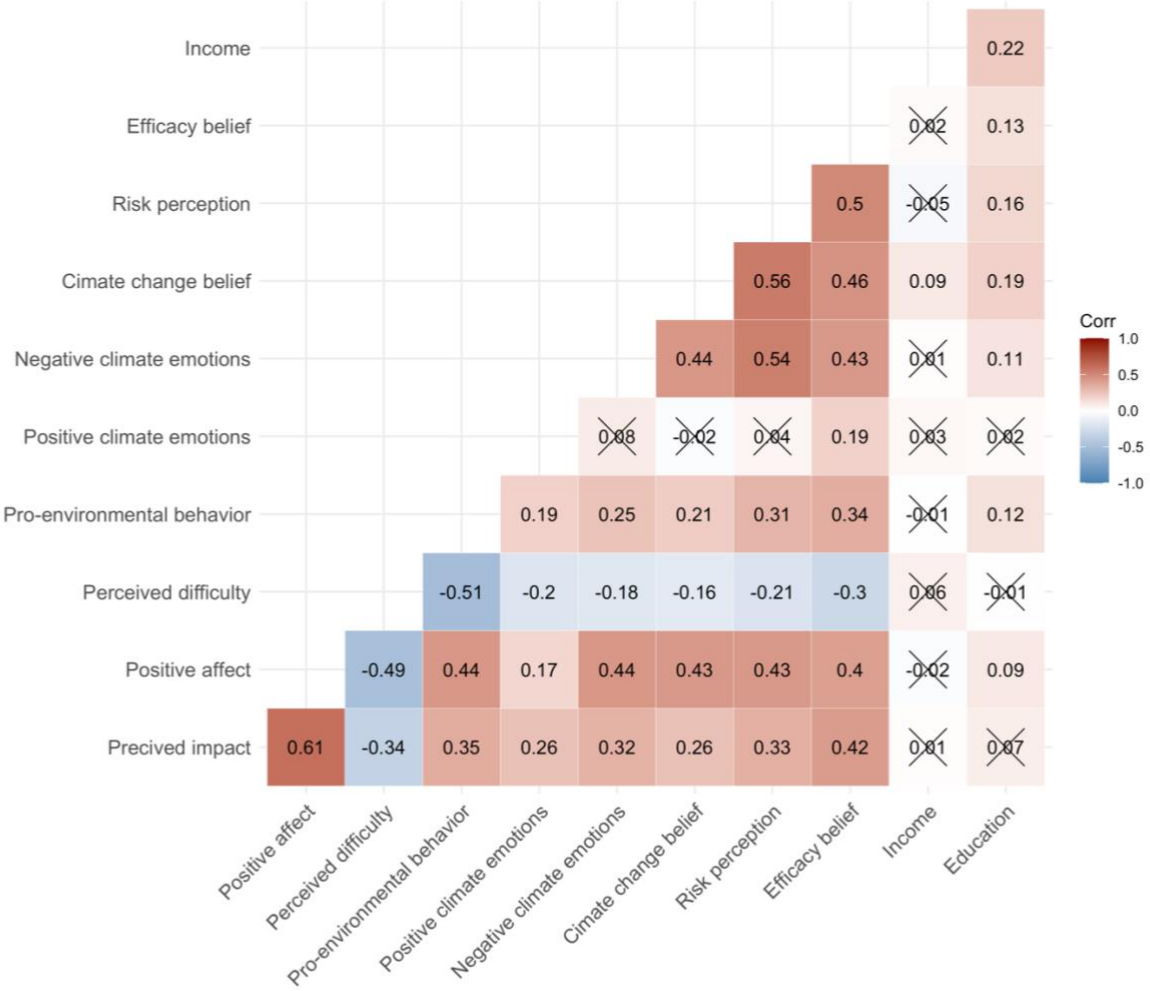


**Figure S2.** Spearman's rank correlations between survey variables over individuals (N=555 for all). Repeated ratings (i.e., affect, impact, difficulty, and pro-environmental behavior) are averaged within individuals, Related to Figure 1 and STAR Methods.

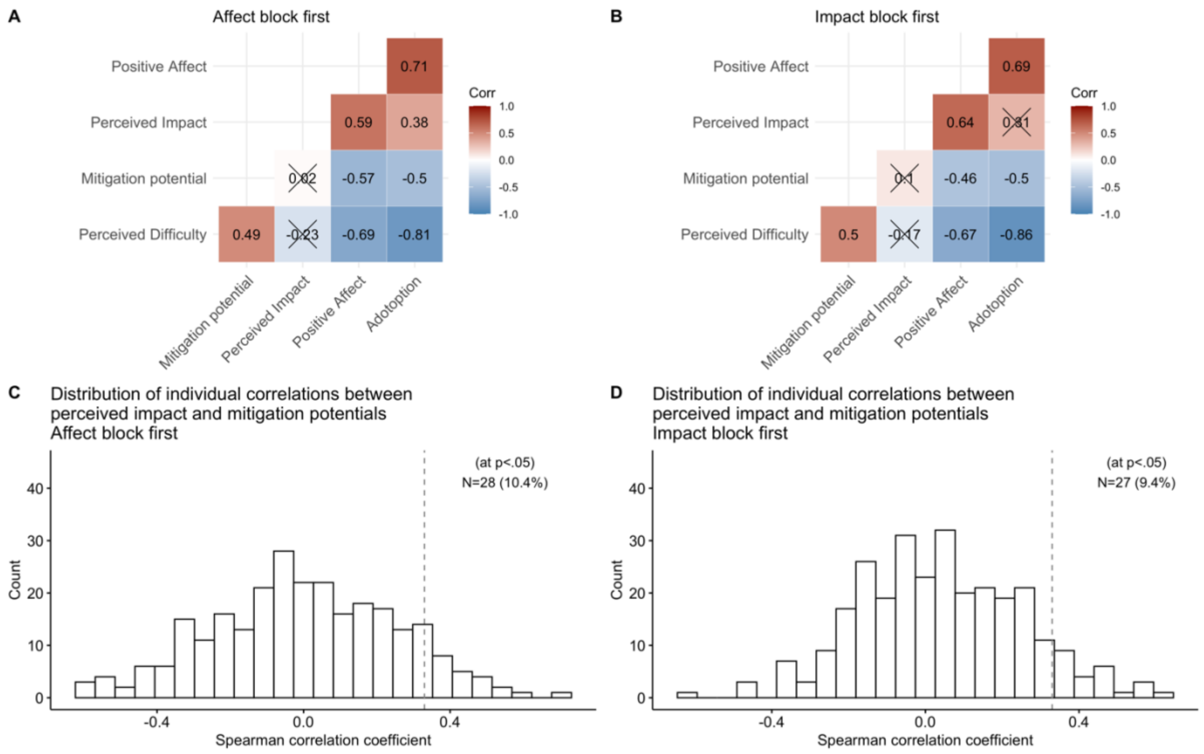


**Figure S3.** Spearman's rank correlations between perception of mitigative behaviors, estimated mitigation potentials, and adoption rate for participants that rated affect first (Panel A; N=268) and those that rated impact first (Panel B; N=287). The distribution of individual correlations between impact judgments and the estimated mitigation potentials for different block orders (Affect-first in Panel C; impact-first in Panel D). Related to Figure 1 and STAR Methods.
